# (*Z*)-1-[4-Fluoro-2-(pyrrolidin-1-yl)phen­yl]-3-phenyl-2-(1*H*-1,2,4-triazol-1-yl)prop-2-en-1-one

**DOI:** 10.1107/S1600536812018454

**Published:** 2012-05-05

**Authors:** Qin-Mei Wen, Ben-Tao Yin, Cong-Yan Yan, Cheng-He Zhou

**Affiliations:** a Laboratory of Bioorganic & Medicinal Chemistry, School of Chemistry and Chemical Engineering, Southwest University, Chongqing 400715, People’s Republic of China

## Abstract

In the title mol­ecule, C_21_H_19_FN_4_O, the triazole ring forms dihedral angles of 67.0 (1) and 59.6 (1)° with the phenyl and fluoro-substituted benzene rings, respectively. The dihedral angle between the phenyl ring and the fluoro-substituted benzene ring is 79.1 (1)°. The pyrrolidine ring is in a half-chair conformation. In the crystal, weak C—H⋯O and C—H⋯N hydrogen bonds connect mol­ecules into layers parallel to (001).

## Related literature

For clinical uses of triazole compounds, see: Wang & Zhou (2011 ▶); Zhou & Wang (2012 ▶); Chang *et al.* (2011 ▶). For the synthesis, see: Solankee *et al.* (2010 ▶). For related structures, see: Wang *et al.* (2009 ▶); Yan *et al.* (2009 ▶).
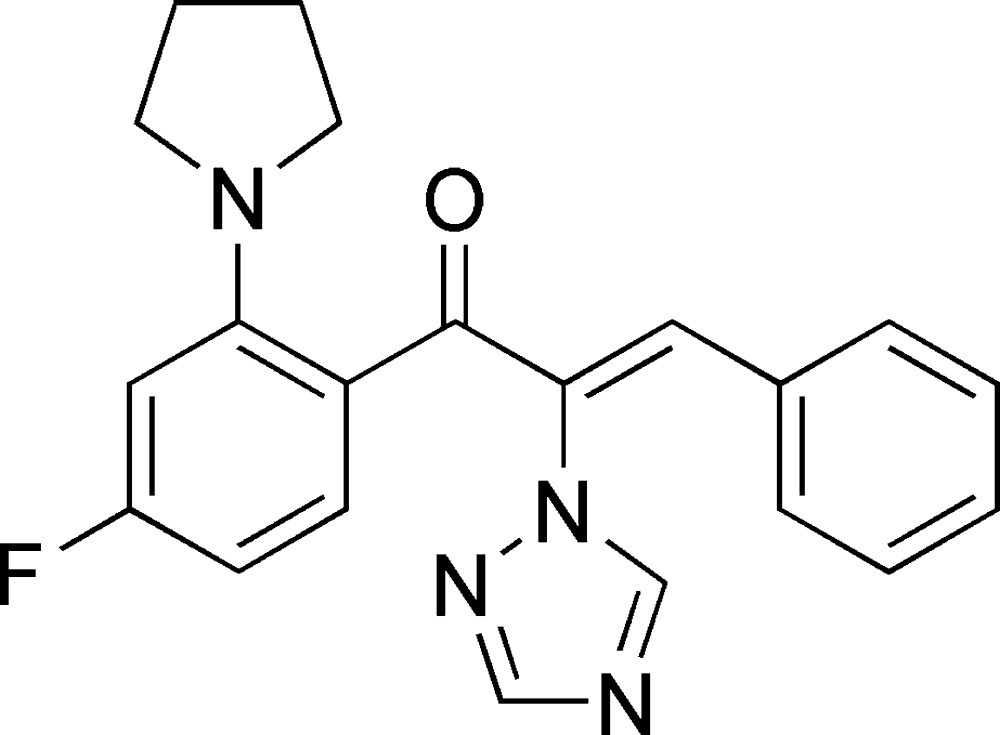


## Experimental

### 

#### Crystal data

C_21_H_19_FN_4_O
*M* *_r_* = 362.40
Monoclinic, 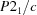

*a* = 11.217 (2) Å
*b* = 10.067 (2) Å
*c* = 15.793 (3) Å
β = 94.47 (3)°
*V* = 1778.0 (6) Å^3^
*Z* = 4
Mo *K*α radiation
μ = 0.09 mm^−1^
*T* = 173 K
0.30 × 0.08 × 0.03 mm

#### Data collection

Bruker SMART CCD diffractometer
Absorption correction: multi-scan (*SADABS*; Sheldrick, 1996) ▶ *T*_min_ = 0.973, *T*_max_ = 0.997
13447 measured reflections
3403 independent reflections
2341 reflections with *I* > 2σ(*I*)
*R*_int_ = 0.058

#### Refinement

*R*[*F* ^2^ > 2σ(*F* ^2^)] = 0.048
*wR*(*F* ^2^) = 0.118
*S* = 1.05
3403 reflections
321 parameters
2 restraints
All H-atom parameters refined
Δρ_max_ = 0.23 e Å^−3^
Δρ_min_ = −0.19 e Å^−3^

### 

Data collection: *SMART* (Bruker, 1997 ▶); cell refinement: *SAINT* (Bruker, 1997 ▶); data reduction: *SAINT*; program(s) used to solve structure: *SHELXS97* (Sheldrick, 2008 ▶); program(s) used to refine structure: *SHELXL97* (Sheldrick, 2008 ▶); molecular graphics: *PLATON* (Spek, 2009 ▶); software used to prepare material for publication: *SHELXTL* (Sheldrick, 2008 ▶).

## Supplementary Material

Crystal structure: contains datablock(s) global, I. DOI: 10.1107/S1600536812018454/lh5432sup1.cif

Structure factors: contains datablock(s) I. DOI: 10.1107/S1600536812018454/lh5432Isup2.hkl

Supplementary material file. DOI: 10.1107/S1600536812018454/lh5432Isup3.cml

Additional supplementary materials:  crystallographic information; 3D view; checkCIF report

## Figures and Tables

**Table 1:** Hydrogen-bond geometry (Å, °)

*D*—H⋯*A*	*D*—H	H⋯*A*	*D*⋯*A*	*D*—H⋯*A*
C5—H12⋯O1^i^	0.981 (19)	2.435 (19)	3.347 (2)	154.6 (14)
C16—H23⋯N1^ii^	0.906 (19)	2.525 (19)	3.388 (3)	159.3 (16)

Symmetry codes: (i) 
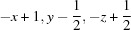
; (ii) 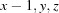.

**Fig. 1.**
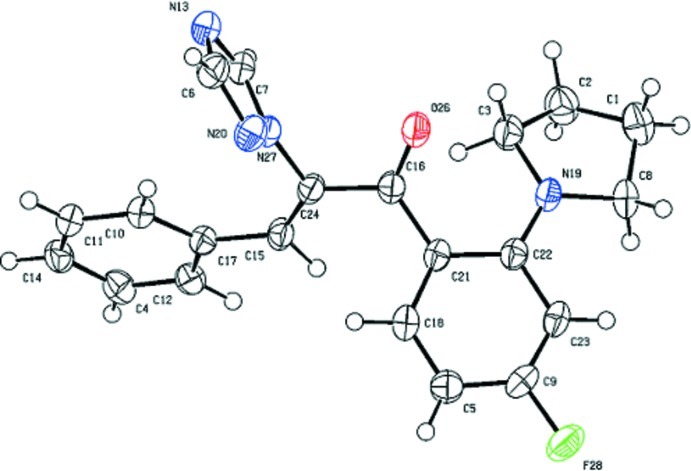
Ellipsoid plot.

## supplementary crystallographic information

### Comment

Chalcones are an important type of biologically active compounds with a diarylenone structural unit (Solankee *et al.*, 2010). Triazole compounds have been shown to have clinical uses (Wang *et al.*, 2011; Zhou *et al.*, 2012; Chang *et al.*, 2011). Our group have been contributing to the research and development of triazolyl chalcones as potential antimicrobial agents. Related structures of triazolylchalcones have alreay been reported (Wang *et al.*, 2009; Yan *et al.*, 2009). Herein we report the crystal structure of the title compound (I).

In the molecular structure (Fig. 1) the triazole ring [N1/N2/N3/C9/C10] forms dihedral angles of 67.0 (1) and 59.6 (1)° with the phenyl [C1-C6] and fluoro-substituted benzene [C12-C17] rings. The dihedral angles between the phenyl and fluoro-substituted bezene rings is 79.1 (1)°. The pyrrolidine ring [N4/C18-C21] is in a half-chair conformation. In the crystal, weak C—H···O and C—H···N hydrogen bonds connect molecules into layers parallel to (001).

### Experimental

The title compound (I) was synthesized according to the procedure of Solankee *et al.* (2010). To a stirring mixture of 1-(2,4-difluorophenyl)-2-(1H-1,2,4-triazol-1-yl)ethanone (2.23 g, 10 mmol) and benzaldehyde (1.06 g, 10 mmol) in ethanol (20 mL) in the presence of acetic acid (0.08 mL, 1.4 mmol) was added pyrrolidine (0.71 g, 10 mmol). The mixed solution was refluxed until the reaction came to the end (monitored by TLC). Subsequently, the solvent was removed under reduced pressure, and the residue was dissolved in dichloromethane (20 mL) and extracted with water (3 x 20 mL). The combined organic phase was dried over anhydrous sodium sulfate and concentrated under reduced pressure to produce the crude product, which was purified by silica gel column chromatography eluting with petroleum ether/ethyl acetate (15/1:1/1, V/V) to afford the desired compound. A crystal suitable for X-ray analysis was grown from a solution of (I) in petroleum ether and ethyl acetate by slow evaporation at room temperature.

### Refinement

All hydrogen atoms were refined independently with isotropic displacement parameters.

### Crystal data

**Table:**

C_21_H_19_FN_4_O	*Z* = 4
*M**_r_* = 362.40	*F*(000) = 760
Monoclinic, *P*2_1_/*c*	*D*_x_ = 1.354 Mg m^−^^3^
Hall symbol: -P 2ybc	Mo *K*α radiation, λ = 0.71073 Å
*a* = 11.217 (2) Å	θ = 2.3–27.7°
*b* = 10.067 (2) Å	µ = 0.09 mm^−^^1^
*c* = 15.793 (3) Å	*T* = 173 K
β = 94.47 (3)°	Plate, yellow
*V* = 1778.0 (6) Å^3^	0.30 × 0.08 × 0.03 mm

### Data collection

**Table:**

Bruker SMART CCD diffractometer	3403 independent reflections
Radiation source: fine-focus sealed tube	2341 reflections with *I* > 2σ(*I*)
Graphite monochromator	*R*_int_ = 0.058
φ and ω scans	θ_max_ = 26.0°, θ_min_ = 3.1°
Absorption correction: multi-scan (*SADABS*; Sheldrick, 1996)	*h* = −13→13
*T*_min_ = 0.973, *T*_max_ = 0.997	*k* = −11→12
13447 measured reflections	*l* = −19→19

### Refinement

**Table:**

Refinement on *F*^2^	Secondary atom site location: difference Fourier map
Least-squares matrix: full	Hydrogen site location: inferred from neighbouring sites
*R*[*F*^2^ > 2σ(*F*^2^)] = 0.048	All H-atom parameters refined
*wR*(*F*^2^) = 0.118	*w* = 1/[σ^2^(*F*_o_^2^) + (0.0576*P*)^2^] where *P* = (*F*_o_^2^ + 2*F*_c_^2^)/3
*S* = 1.05	(Δ/σ)_max_ < 0.001
3403 reflections	Δρ_max_ = 0.23 e Å^−^^3^
321 parameters	Δρ_min_ = −0.19 e Å^−^^3^
2 restraints	Extinction correction: *SHELXL97* (Sheldrick, 2008), Fc^*^=kFc[1+0.001xFc^2^λ^3^/sin(2θ)]^-1/4^
Primary atom site location: structure-invariant direct methods	Extinction coefficient: 0.0075 (13)

### Special details

**Table:**

Geometry. All e.s.d.'s (except the e.s.d. in the dihedral angle between two l.s. planes) are estimated using the full covariance matrix. The cell e.s.d.'s are taken into account individually in the estimation of e.s.d.'s in distances, angles and torsion angles; correlations between e.s.d.'s in cell parameters are only used when they are defined by crystal symmetry. An approximate (isotropic) treatment of cell e.s.d.'s is used for estimating e.s.d.'s involving l.s. planes.
Refinement. Refinement of *F*^2^ against ALL reflections. The weighted *R*-factor *wR* and goodness of fit *S* are based on *F*^2^, conventional *R*-factors *R* are based on *F*, with *F* set to zero for negative *F*^2^. The threshold expression of *F*^2^ > σ(*F*^2^) is used only for calculating *R*-factors(gt) *etc*. and is not relevant to the choice of reflections for refinement. *R*-factors based on *F*^2^ are statistically about twice as large as those based on *F*, and *R*- factors based on ALL data will be even larger.

### Fractional atomic coordinates and isotropic or equivalent isotropic displacement parameters (Å^2^)

**Table:**

	*x*	*y*	*z*	*U*_iso_*/*U*_eq_	
F1	0.06331 (9)	0.07121 (12)	0.14879 (7)	0.0506 (4)	
N3	0.73647 (12)	−0.01216 (15)	0.27605 (9)	0.0291 (4)	
O1	0.54633 (11)	0.01888 (14)	0.36550 (8)	0.0389 (4)	
C8	0.62355 (15)	−0.06156 (19)	0.24237 (11)	0.0289 (4)	
C16	0.18387 (18)	−0.0239 (2)	0.25987 (13)	0.0340 (5)	
C17	0.29747 (16)	−0.06191 (18)	0.29689 (11)	0.0290 (4)	
C12	0.39893 (16)	−0.02771 (18)	0.25277 (11)	0.0290 (4)	
N2	0.76525 (14)	0.11901 (16)	0.27051 (10)	0.0378 (4)	
N4	0.30375 (13)	−0.13062 (16)	0.37147 (9)	0.0320 (4)	
C13	0.38080 (18)	0.02722 (19)	0.17088 (12)	0.0336 (5)	
C6	0.70879 (16)	−0.19160 (19)	0.12221 (11)	0.0291 (4)	
C11	0.52247 (16)	−0.02255 (19)	0.29265 (12)	0.0306 (5)	
C7	0.61641 (17)	−0.14582 (19)	0.17621 (11)	0.0312 (5)	
C3	0.87605 (18)	−0.2952 (2)	0.01763 (12)	0.0369 (5)	
N1	0.91459 (14)	0.00330 (19)	0.34166 (10)	0.0422 (5)	
C5	0.68283 (18)	−0.3030 (2)	0.07170 (11)	0.0324 (5)	
C2	0.90214 (18)	−0.1816 (2)	0.06478 (12)	0.0369 (5)	
C1	0.82010 (17)	−0.1292 (2)	0.11679 (12)	0.0330 (5)	
C15	0.17490 (16)	0.0344 (2)	0.18190 (13)	0.0363 (5)	
C18	0.19584 (18)	−0.1755 (2)	0.41003 (13)	0.0387 (5)	
C10	0.82587 (17)	−0.0775 (2)	0.31872 (12)	0.0354 (5)	
C9	0.87283 (19)	0.1214 (2)	0.31018 (13)	0.0406 (5)	
C14	0.26982 (18)	0.0594 (2)	0.13393 (14)	0.0365 (5)	
C4	0.76540 (19)	−0.3541 (2)	0.01975 (12)	0.0365 (5)	
C21	0.40905 (18)	−0.2063 (2)	0.40537 (14)	0.0392 (5)	
C20	0.3575 (2)	−0.3164 (2)	0.45719 (15)	0.0446 (6)	
C19	0.2455 (2)	−0.2535 (3)	0.48764 (15)	0.0477 (6)	
H15	0.5351 (16)	−0.1852 (18)	0.1620 (10)	0.031 (5)*	
H23	0.1166 (17)	−0.0388 (19)	0.2867 (12)	0.038 (6)*	
H3A	0.4540 (15)	−0.2398 (18)	0.3554 (11)	0.037 (5)*	
H5	0.2578 (17)	0.1005 (19)	0.0796 (12)	0.042 (6)*	
H12	0.6041 (17)	−0.3451 (19)	0.0728 (11)	0.036 (5)*	
H7	0.8209 (17)	−0.174 (2)	0.3276 (12)	0.044 (6)*	
H8A	0.1441 (17)	−0.096 (2)	0.4266 (11)	0.043 (6)*	
H2A	0.3393 (17)	−0.396 (2)	0.4217 (12)	0.044 (6)*	
H18	0.4500 (16)	0.0451 (18)	0.1429 (12)	0.037 (5)*	
H6	0.9173 (18)	0.205 (2)	0.3160 (12)	0.048 (6)*	
H4	0.7450 (17)	−0.432 (2)	−0.0150 (12)	0.041 (6)*	
H14	0.9352 (18)	−0.332 (2)	−0.0180 (11)	0.047 (6)*	
H11	0.9777 (18)	−0.1339 (19)	0.0632 (11)	0.039 (5)*	
H8B	0.1457 (18)	−0.2321 (19)	0.3678 (12)	0.046 (6)*	
H10	0.8385 (17)	−0.0451 (19)	0.1478 (12)	0.044 (6)*	
H3B	0.4656 (19)	−0.144 (2)	0.4417 (13)	0.057 (7)*	
H1A	0.1847 (19)	−0.322 (2)	0.5052 (13)	0.060 (7)*	
H2B	0.4184 (19)	−0.346 (2)	0.5040 (12)	0.049 (6)*	
H1B	0.267 (2)	−0.193 (2)	0.5352 (14)	0.068 (8)*	

### Atomic displacement parameters (Å^2^)

**Table:**

	*U*^11^	*U*^22^	*U*^33^	*U*^12^	*U*^13^	*U*^23^
F1	0.0285 (7)	0.0611 (9)	0.0608 (8)	0.0060 (6)	−0.0043 (6)	0.0095 (6)
N3	0.0224 (8)	0.0345 (10)	0.0308 (8)	−0.0006 (7)	0.0053 (7)	−0.0009 (7)
O1	0.0283 (8)	0.0522 (10)	0.0372 (8)	−0.0007 (7)	0.0075 (6)	−0.0078 (7)
C8	0.0218 (10)	0.0320 (11)	0.0329 (10)	0.0000 (8)	0.0030 (8)	0.0016 (9)
C16	0.0223 (10)	0.0366 (12)	0.0439 (12)	−0.0043 (9)	0.0078 (9)	−0.0025 (10)
C17	0.0254 (10)	0.0299 (11)	0.0322 (10)	−0.0002 (8)	0.0048 (8)	−0.0047 (8)
C12	0.0236 (10)	0.0302 (11)	0.0337 (10)	0.0025 (8)	0.0058 (8)	−0.0019 (8)
N2	0.0318 (10)	0.0360 (10)	0.0458 (10)	−0.0048 (8)	0.0035 (8)	−0.0013 (8)
N4	0.0231 (8)	0.0370 (10)	0.0367 (9)	0.0018 (7)	0.0083 (7)	−0.0001 (7)
C13	0.0277 (11)	0.0356 (12)	0.0387 (11)	−0.0006 (9)	0.0098 (10)	0.0000 (9)
C6	0.0257 (10)	0.0330 (11)	0.0288 (10)	0.0029 (9)	0.0035 (8)	0.0026 (8)
C11	0.0268 (10)	0.0327 (11)	0.0329 (11)	0.0002 (9)	0.0066 (9)	0.0015 (9)
C7	0.0235 (10)	0.0355 (12)	0.0355 (11)	−0.0020 (9)	0.0070 (9)	0.0044 (9)
C3	0.0363 (12)	0.0447 (13)	0.0306 (10)	0.0121 (10)	0.0084 (9)	0.0003 (10)
N1	0.0268 (9)	0.0613 (13)	0.0389 (10)	−0.0053 (9)	0.0053 (8)	0.0048 (9)
C5	0.0337 (11)	0.0331 (12)	0.0308 (10)	−0.0020 (10)	0.0042 (9)	0.0042 (9)
C2	0.0249 (11)	0.0521 (14)	0.0341 (11)	0.0002 (10)	0.0048 (9)	0.0006 (10)
C1	0.0272 (11)	0.0398 (12)	0.0325 (10)	0.0008 (9)	0.0060 (9)	−0.0008 (10)
C15	0.0219 (10)	0.0383 (12)	0.0480 (12)	0.0018 (9)	−0.0028 (9)	−0.0021 (10)
C18	0.0315 (11)	0.0439 (14)	0.0429 (12)	−0.0019 (10)	0.0172 (10)	0.0001 (10)
C10	0.0260 (11)	0.0468 (14)	0.0340 (11)	0.0029 (10)	0.0068 (9)	0.0041 (10)
C9	0.0330 (12)	0.0464 (14)	0.0427 (12)	−0.0101 (11)	0.0049 (10)	−0.0016 (11)
C14	0.0358 (12)	0.0367 (12)	0.0371 (12)	0.0028 (10)	0.0032 (10)	0.0045 (10)
C4	0.0436 (13)	0.0345 (13)	0.0319 (11)	0.0027 (10)	0.0069 (10)	−0.0014 (10)
C21	0.0328 (12)	0.0448 (14)	0.0409 (12)	0.0070 (11)	0.0086 (10)	0.0048 (11)
C20	0.0460 (14)	0.0441 (15)	0.0447 (13)	0.0036 (12)	0.0099 (11)	0.0071 (12)
C19	0.0506 (15)	0.0478 (15)	0.0471 (13)	−0.0030 (12)	0.0187 (12)	0.0058 (12)

### Geometric parameters (Å, º)

**Table:**

F1—C15	1.370 (2)	C3—H14	0.974 (19)
N3—C10	1.337 (2)	N1—C10	1.314 (3)
N3—N2	1.364 (2)	N1—C9	1.358 (3)
N3—C8	1.425 (2)	C5—C4	1.383 (3)
O1—C11	1.233 (2)	C5—H12	0.980 (19)
C8—C7	1.343 (3)	C2—C1	1.385 (3)
C8—C11	1.487 (2)	C2—H11	0.976 (19)
C16—C15	1.361 (3)	C1—H10	0.99 (2)
C16—C17	1.413 (3)	C15—C14	1.377 (3)
C16—H23	0.906 (19)	C18—C19	1.524 (3)
C17—N4	1.363 (2)	C18—H8A	1.03 (2)
C17—C12	1.422 (2)	C18—H8B	1.01 (2)
C12—C13	1.407 (3)	C10—H7	0.98 (2)
C12—C11	1.478 (3)	C9—H6	0.98 (2)
N2—C9	1.316 (3)	C14—H5	0.954 (19)
N4—C18	1.468 (2)	C4—H4	0.98 (2)
N4—C21	1.471 (2)	C21—C20	1.519 (3)
C13—C14	1.372 (3)	C21—H3A	1.026 (18)
C13—H18	0.940 (19)	C21—H3B	1.03 (2)
C6—C5	1.394 (3)	C20—C19	1.519 (3)
C6—C1	1.406 (3)	C20—H2A	0.99 (2)
C6—C7	1.467 (2)	C20—H2B	1.01 (2)
C7—H15	1.004 (18)	C19—H1A	1.03 (2)
C3—C4	1.378 (3)	C19—H1B	0.98 (2)
C3—C2	1.384 (3)		
			
C10—N3—N2	109.69 (16)	C2—C1—H10	119.7 (11)
C10—N3—C8	128.79 (17)	C6—C1—H10	120.2 (11)
N2—N3—C8	121.47 (15)	C16—C15—F1	117.59 (17)
C7—C8—N3	120.55 (16)	C16—C15—C14	124.80 (18)
C7—C8—C11	125.48 (17)	F1—C15—C14	117.60 (17)
N3—C8—C11	113.65 (15)	N4—C18—C19	103.35 (17)
C15—C16—C17	119.48 (19)	N4—C18—H8A	111.4 (10)
C15—C16—H23	119.3 (12)	C19—C18—H8A	111.9 (10)
C17—C16—H23	121.2 (12)	N4—C18—H8B	109.5 (11)
N4—C17—C16	118.64 (16)	C19—C18—H8B	112.8 (11)
N4—C17—C12	123.83 (16)	H8A—C18—H8B	107.9 (15)
C16—C17—C12	117.52 (17)	N1—C10—N3	110.9 (2)
C13—C12—C17	118.80 (17)	N1—C10—H7	128.5 (12)
C13—C12—C11	116.20 (16)	N3—C10—H7	120.6 (12)
C17—C12—C11	124.01 (16)	N2—C9—N1	115.8 (2)
C9—N2—N3	101.55 (17)	N2—C9—H6	120.1 (12)
C17—N4—C18	121.73 (16)	N1—C9—H6	124.1 (12)
C17—N4—C21	124.29 (15)	C13—C14—C15	115.96 (19)
C18—N4—C21	110.76 (15)	C13—C14—H5	123.1 (12)
C14—C13—C12	123.04 (19)	C15—C14—H5	120.9 (12)
C14—C13—H18	120.7 (11)	C3—C4—C5	120.1 (2)
C12—C13—H18	116.2 (11)	C3—C4—H4	120.3 (11)
C5—C6—C1	118.15 (17)	C5—C4—H4	119.6 (11)
C5—C6—C7	117.39 (17)	N4—C21—C20	104.23 (17)
C1—C6—C7	124.45 (18)	N4—C21—H3A	108.6 (10)
O1—C11—C12	122.52 (16)	C20—C21—H3A	113.9 (10)
O1—C11—C8	117.93 (16)	N4—C21—H3B	109.3 (12)
C12—C11—C8	119.47 (16)	C20—C21—H3B	112.6 (12)
C8—C7—C6	130.44 (18)	H3A—C21—H3B	108.0 (15)
C8—C7—H15	114.9 (10)	C21—C20—C19	103.03 (19)
C6—C7—H15	114.6 (10)	C21—C20—H2A	110.8 (11)
C4—C3—C2	119.69 (19)	C19—C20—H2A	112.1 (12)
C4—C3—H14	120.5 (12)	C21—C20—H2B	110.3 (12)
C2—C3—H14	119.8 (12)	C19—C20—H2B	114.9 (12)
C10—N1—C9	102.06 (18)	H2A—C20—H2B	105.9 (17)
C4—C5—C6	121.24 (19)	C20—C19—C18	102.69 (18)
C4—C5—H12	119.7 (11)	C20—C19—H1A	112.8 (12)
C6—C5—H12	119.1 (11)	C18—C19—H1A	110.9 (12)
C3—C2—C1	120.8 (2)	C20—C19—H1B	110.2 (14)
C3—C2—H11	122.7 (11)	C18—C19—H1B	110.3 (14)
C1—C2—H11	116.5 (12)	H1A—C19—H1B	109.8 (18)
C2—C1—C6	120.0 (2)		

### Hydrogen-bond geometry (Å, º)

**Table:**

*D*—H···*A*	*D*—H	H···*A*	*D*···*A*	*D*—H···*A*
C5—H12···O1^i^	0.981 (19)	2.435 (19)	3.347 (2)	154.6 (14)
C16—H23···N1^ii^	0.906 (19)	2.525 (19)	3.388 (3)	159.3 (16)

Symmetry codes: (i) −*x*+1, *y*−1/2, −*z*+1/2; (ii) *x*−1, *y*, *z*.
